# *C14orf132* gene is possibly related to extremely low birth weight

**DOI:** 10.1186/s12863-016-0439-5

**Published:** 2016-09-22

**Authors:** Airi Tiirats, Triin Viltrop, Margit Nõukas, Ene Reimann, Andres Salumets, Sulev Kõks

**Affiliations:** 1 Department of Obstetrics and Gynaecology, University of Tartu, 8 Puusepa Street, 51014 Tartu, Estonia; 2 Competence Centre on Health Technologies, Tartu, Estonia; 3 Institute of Biomedicine and Translational Medicine, University of Tartu, Tartu, Estonia; 4 Institute of Molecular and Cell Biology, University of Tartu, Tartu, Estonia; 5 Estonian Genome Centre, University of Tartu, Tartu, Estonia; 6 Core Facility of Clinical Genomics, University of Tartu, Tartu, Estonia; 7 Department of Pathophysiology, Institute of Biomedicine and Translational Medicine, University of Tartu, Tartu, Estonia; 8 Department of Obstetrics and Gynecology, University of Helsinki and Helsinki University Hospital, Helsinki, Finland; 9 Centre of Translational Medicine, University of Tartu, Tartu, Estonia; 10 Department of Reproductive Biology, Estonian University of Life Sciences, Tartu, Estonia

**Keywords:** Copy number variants, *C14orf132*, Extremely low birth weight, RNA-seq and long non-coding RNA

## Abstract

**Background:**

Despite extensive research the genetic component of extremely low birth weight (ELBW) in newborns has remained obscure.

**Results:**

The aim of the case study was to identify candidate gene(s) causing ELBW in newborns and hypotrophy in infants. A family of four was studied: mother, father and two ELBW-phenotype children. Studies were made of the medical conditions of the second child at birth and post-partum - peculiar phenotype*,* micro-anomalies, recurrent infections, suspicion of autoimmune hepatitis, multifactorial encephalopathy and suspected metabolic and chromosomal abnormalities. Whole genome single nucleotide polymorphism (SNP) genotyping array was used to investigate the genomic rearrangements in both affected children using peripheral blood DNA samples. Whole blood transcriptome was assessed by using RNA sequencing (RNA-seq) in all four family members. RNA-seq identified a single gene – *C14orf132* (chromosome 14 open reading frame 132) differentially expressed, with the level of the transcript significantly lower in the blood samples of the children. Copy number variant (CNV) analysis did not reveal any pathogenic CNVs in the region of *C14orf132* gene of both affected children.

**Conclusion:**

We demonstrated the importance of combining whole genome CNV and transcriptome analysis in identification of the candidate gene(s) in case studies. We propose the *C14orf132* gene expression to be associated with the ELBW-phenotype. *C14orf132* gene is a novel long non-coding RNA (lincRNA) with unknown function, which might be associated with the pre- and early postnatal developmental delay through the altered gene expression.

**Electronic supplementary material:**

The online version of this article (doi:10.1186/s12863-016-0439-5) contains supplementary material, which is available to authorized users.

## Background

Preterm birth is a major cause of morbidity and mortality among newborns. Complications of preterm birth are the largest cause of neonatal deaths, responsible for 35 % of the 3.1 million deaths a year worldwide [1]. Population-based cohort studies of low birth weight infants have shown that reduced birth weight is associated with a wide range of health, cognitive and behavioural difficulties in child- and adulthood [2]. Lower birth weight is even associated with increased all-cause mortality at all ages among adult women [3]. These adverse sequelae are thought to arise via epigenetic mechanisms of foetal and placental development. In addition, genetic factors have also shown to play a pivotal role in the aetiology of low birth weight, supporting the hypothesis that genetic variations, like those in insulin-like growth factor-I gene, could account for the association between low birth weight and susceptibility to diabetes and cardiovascular disease in later life [4]. All this aforementioned explains why the genetic origin of low birth weight deserves more attention.

In the current case study we describe a family with two children with ELBW. Whole blood transcriptome RNA-seq analysis and whole genome SNP genotyping array to investigate CNVs were performed to find potential genetic factors involved in the ELBW phenotype.

## Methods

### Materials and analysis

The proband (Child A) is the second child (female) of a non-consanguineous couple and was 7 months old at time of death. Blood samples from Child A were collected post-mortem. The family’s first child (male) (Child B) was 4 years old by the time of analysis.

Peripheral blood for RNA extraction was collected into Tempus Blood RNA Tubes (Ambion, Life Technologies) and RNA was isolated using Tempus Spin RNA Isolation Kit (Ambion). The whole transcriptome RNA-seq libraries were constructed with SOLiD Total RNA-seq Kit (Ambion). 75 bp in forward direction was sequenced using SOLiD 5500 W platform (Life Technologies). RNA-seq raw data were mapped using LifeScope software (Applied Biosystems, Thermo Fisher Scientific) to the human reference genome (hg19) and raw counts were analysed with R statistical software using edgeR package [5].

Quantitative real-time (qRT)-PCR was used to validate the RNA-seq results of all four family members. In addition, ten control family trios with appropriate for gestational age (AGA) newborn (average ± SD: birth weight 3684 ± 467 g and gestational age 39 weeks 6 days ± 1 week 3 days), and five trios and four mother-child duos with small for gestational age (SGA) newborns (birth weight 1920 ± 738 g and gestational age 38 weeks 3 days ± 2 weeks 1 day) were also analysed. Samples added for validation were extracted from the maternal and paternal peripheral and newborns’ umbilical cord blood.

SNP genotyping experiment was performed on genomic DNA extracted from peripheral blood of both siblings. Samples were genotyped for 542,585 markers using the Infinium HumanCoreExome BeadChip (Illumina Inc). Genotypes were called by GenomeStudio software Genotyping Module v.3.1 (Illumina Inc.). Log R Ratio (LRR) and B Allele Frequency (BAF) values generated by the GenomeStudio software were used for CNV calling using Hidden Markov Model-based software PennCNV [6].

### Family presentation

Child A was mothers’ second child from her second complicated pregnancy. The mother suffered from a respiratory viral infection (not treated) on the 17th week of the pregnancy. During weeks 19–21 foetal growth retardation was diagnosed and at the 22nd week of pregnancy decrease in amniotic fluid was seen in ultrasound (US). TORCH test was performed on maternal peripheral blood and it was negative for infections. The amniotic fluid chromosomal testing revealed a normal female-foetus with karyotype of 46, XX. Maternal laparoscopic cholecystectomy was performed on the 27th week of pregnancy due to a gall bladder infection.

As a result of reduced uteroplacental blood flow (UBF) of 3rd class and maternal infection, emergency caesarean section was performed at gestational age of 27 weeks and 4 days. Child A Apgar scores were 6, 7 and 8 at 1, 5 and 10 min, respectively. The child was SGA with following measurements: weight 470 g, length 27 cm and head circumference 23 cm. Placenta was very small - 108 g (10th percentile is 192 g), with the umbilical cord positioned distally. Placental histological study results of child “A” and “B” were highly similar demonstrating areas of ischemic infarction, decidual vasculopathy, calcification of infarction zone, subcortical fibrinoid deposits and non-inflammatory pattern. Moreover, even weight of placenta was identical.

The newborn was intubated immediately after birth due to respiratory failure. Subsequent problems were liver failure (persistent hyperbilirubinemia, elevated alkaline phosphatase levels and hepatosplenomegaly) and coagulopathy (thrombocytopenia, low antithrombin II activity, low protein C and S and lower than normal prothrombin).

Medical geneticists’ consultation was obtained at postnatal age of 1 month and 16 days (corrected age of 35 weeks) due to peculiar phenotype – extremities and a small trunk compared to the head and micro-anomalies (mongoloid eye shape, long inner canthal distance, low glabella, large tongue, high forehead, and small mandible). Due to the unexpected course of the disease, metabolic diseases and chromosomal abnormalities were suspected. However, genetic testing, including the disease-associated mutations in the cystic fibrosis transmembrane conductance regulator gene, alpha-1-antithrypsin deficiency (*SERPINA1* S and Z alleles), galactosemia (*GALT* gene mutations) and imprinting disorders within Silver–Russell syndrome region at 11p15.5 were found to be normal.

Despite of intensive neonatal care the proband died from pulmocardiac insufficiency at age 7 months and 7 days.

Family history revealed that the father of Child A was healthy and the proband was his first child. Mother (height 160 cm and weight approximately 110 kg) had no previous miscarriages. She was tested for antinuclear antibodies (1:10, neg.), gastric parietal cell antibodies (1:100, pos.), anticardiolipin antibodies (<12 RU/ml, neg.), beta-2 glycoprotein 1 antibodies (neg.), protein C (70–130 %) and free protein S (60–140 %). Thrombophilia was not confirmed for the mother.

Child B, who was also born via emergency caesarean section at 30 weeks of gestation with birth weight of 660 g and Apgar scores of 4, 7 and 7 at 1, 5 and 10 min, respectively. Similarly before delivery, reduced UBF of 3rd class was diagnosed, with almost no detection of amniotic fluid with US and a small placenta (109 g) at delivery. Child B was intubated shortly after birth due to respiratory failure caused by congenital sepsis and quickly followed by deterioration into a septic shock. Subsequent problems were perinatal bronchopulmonary dysplasia, feeding disorders and other pathologies. The child left the hospital at 2.5 months of age and weighed 2560 g at the time with constant mild respiratory disorders and cerebral palsy. At age four, when present study was conducted, Child B had non-allergic asthma and behavioural problems. Father of Child B died in an accident 3 weeks before the birth. Timeline summarizing the clinical course of the children is presented in Additional file 1.

## Results

### RNA-seq and qRT-PCR results

Whole blood transcriptome RNA-seq was used to study gene expression patterns in the subject family, including parents and two children. By comparing transcriptomes of both children to parental gene expression data, six differentially expressed genes with false discovery rate (FDR) of <0.2 were identified (Table 1). The most significant difference was evident for the gene *C14orf132*. In both children, the RNA of *C14orf132* was not detected with RNA-seq. However, both mother and father had detectable RNA levels in their blood – the read counts were 29 and 16, respectively.

**Table 1:** RNA-seq results of the blood RNA of ELBW children and their parents

Symbol	FC	log_2_FC	log_2_CPM	*P*-value	False discovery rate	Chr	Entrez gene name
*C14orf132*	0.006	−7.42	2.28	3.42E-07	0.008	chr14	Chromosome 14 open reading frame 132
*HBG2*	112.985	6.82	11.02	1.62E-05	0.130	chr11	Hemoglobin, gamma A
*HIST1H3C*	6.634	2.73	3.59	2.24E-05	0.130	chr6	Histone cluster 1, H3a
*PTPN13*	0.150	−2.74	3.32	1.77E-05	0.130	chr4	Protein tyrosine phosphatase, non-receptor type 12
*ARG1*	23.263	4.54	6.18	5.14E-05	0.198	chr6	Arginase 1
*IGLL5*	7.945	2.99	6.67	4.81E-05	0.198	chr22	Immunoglobulin lambda-like polypeptide 3, pseudogene

*FC* fold change, *CPM* counts per million, *Chr* chromosome

**Fig. 1:**
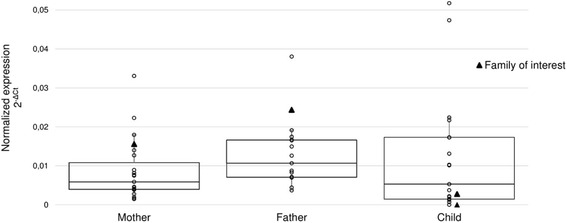
Relative *C14orf132* gene expression in controls and family of interest as measured by RT-PCR. No statistically significant differences between the groups were detected

qRT-PCR analysis using TaqMan assay confirmed higher parental expression level of *C14orf132* in the subject family. Thus, the gene expression analysis with two independent methods identified significantly downregulated *C14orf132* gene expression in ELBW-children, when compared to their parents. However, the difference in *C14orf132* gene expression was found to be unique for the subject family, as similar gene expression was shown for parents and children in control families (Fig. 1).

### CNV analysis results

The whole genome CNV analysis of Child A revealed 473 kb duplication in chromosome region 12p11.23 (chr12: 27,315,291-27,788,795; GRCh37/hg19). The duplicated region contained four genes: *STK38L*, *ARNTL2*, *SMCO2* and *PPFIBP1.* Although the chromosomal gains in 12p11.23 region have been described in the DECIPHER database [7]; no association with any clinical phenotype has been made.

In Child B a 204 kb deletion in 10p15.3 (chr10:1,307,456-1,511,786 GRCh37/hg19) was identified. This deletion included partially deleted *ADARB2* gene, which was unique to Child B and was not identified in Child A. Again, no association with any clinical phenotype has been made.

As no common CNVs were demonstrated for both siblings, the unique CNVs found for Child A (duplication in 12p11.23) and Child B (deletion in 10p15.3) were unlikely involved in the pathogenesis of ELBW phenotype, which was common to both siblings. Furthermore, no genomic aberrations in the *C14orf132* gene region were seen in neither of the children.

## Discussion

In the current case study the whole genome CNV and blood transcriptome studies were conducted in a family with two ELBW-newborns, in order to find the potential causative gene(s) for the extreme phenotype of the children. The ELBW phenotype in the presented family is likely maternally inherited, because the children were naturally conceived by different fathers, but presented similar ELBW-phenotype. Whole transcriptome sequencing of peripheral blood RNA of the mother, father and two children identified *C14orf132* gene as the only differentially expressed gene with high significance. The candidate gene is located at chromosome 14q32 and is a large intergenic lincRNA with unknown function. Previously it has been reported that *C14orf132* is one of the most significantly downregulated lincRNA in hepatocellular carcinoma cell line [8] and non-small-cell lung carcinoma [9]. Nevertheless its function is unknown and further investigations are needed. The expression of the *C14orf132* gene is described to be the highest in the human brain compared to other tissue types (GTEx Portal database - www.gtexportal.org) [10], however as there is almost no functional data, it is not possible to estimate the true effect of downregulated *C14orf132* on the phenotype and pre- and postnatal developmental delay particularly.

In spite of the relation between *C14orf132* and ELBW-phenotype has not been demonstrated the analysis of available databases (NCBI PheGenI [11], NCBI ClinVar [12], and NCBI dbVar [13]) provided additional supportive evidence for the association of *C14orf132* with ELBW. Namely, according to the NCBI ClinVar database [12], which provides a comprehensive information about the known clinical conditions for every genomic region, the locus harbouring *C14orf132* gene has been claimed to be related to the phenotype of “global developmental delay”. However, these genomic changes – deletions and duplications, usually include a more extensive genomic regions with multiple genes. Thus, the proof that the *C14orf132* gene is related to the phenotype with “global developmental delay” still should be confirmed in future studies.

Genotyping analysis did not reveal any CNVs in the *C14orf132* gene region in either of the children. Therefore it is not possible to associate genomic aberrations in the *C14orf132* locus with the altered gene activity as observed in RNA-seq and qRT-PCR. Consequently, these results suggest that the differences in *C14orf132* expression could be caused rather via the epigenetic mechanisms.

Although the lower expression of *C14orf132* in offspring of subject family observed in RNA-seq was also confirmed by qRT-PCR, similar expression pattern of the candidate gene was not detected in the control group of SGA newborn families. Therefore it was suggested that the low expression of *C14orf132* might be a factor associated with ELBW phenotype only in the subject family. Nevertheless it is important to acknowledge that the SGA newborns used as a reference group had an average birth weight of 1920 ± 738 g and gestational age of 38 weeks and 3 days ± 2 weeks and 1 day. Therefore it was not possible to analyse any other severely preterm children with ELBW phenotype like in the subject family for comparison.

## Conclusions

The current case study demonstrates the importance of combining whole genome CNV and transcriptome analysis in determining the possible genes for rare inherited diseases. A large intergenic non-coding RNA with unknown function – *C14orf132* was identified as a potential gene involved in the development of ELBW-phenotype. The findings of the current study provide additional support to our hypothesis that *C14orf132* is related to the regulation of pre- and early postnatal growth and development. However, our study does not provide functional evidence for the causative link between *C14orf132* and ELBW, and for final proof more detailed experiments are needed to support the presented theory.

## Additional file

Additional file 1: Timeline summarizing the clinical course of the children described in this case report. (PDF 210 kb)

## Abbreviations

AGA: Birth weight appropriate for gestational age
*C14orf132*: Chromosome 14 open reading frame 132
Child A: Second born child (female)
Child B: First born child (male)
CNV: Copy number variants
ELBW: Extremely low birth weight
FDR: False discovery rate
lincRNA: Long non-coding RNA
qRT-PCR: Quantitative real-time polymerase chain reaction
RNA-seq: RNA sequencing
SGA: Birth weight small for gestational age
SNP: Single nucleotide polymorphism
TORCH test: Toxoplasma, Rubella, Cytomegalovirus, Herpes Simplex Virus I and II
UBF: Uteroplacental blood flow
US: Ultrasound
