# Supplementary material for: C14orf132 gene is possibly related to extremely low birth weight
Source: BMC Genet. 2016 Sep 22;17:132. doi: 10.1186/s12863-016-0439-5 (PMC5034552; doi:10.1186/s12863-016-0439-5)

Timeline Picture Created Using Microsoft Word

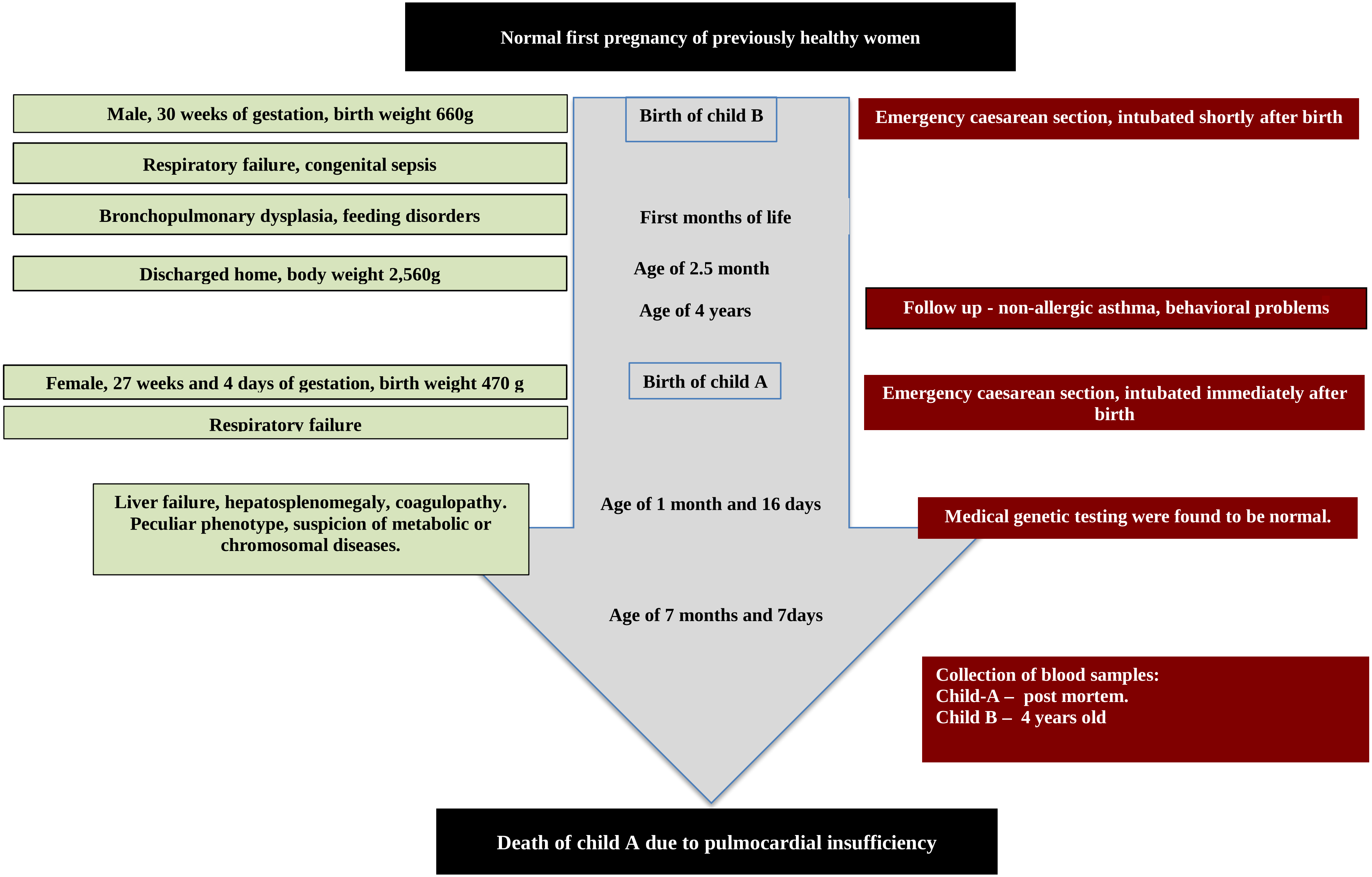

Supplement: Additional file 1: — Timeline summarizing the clinical course of the children described in this case report. (PDF 210 kb) [file 12863_2016_439_MOESM1_ESM.pdf]
